# An Event-related Potential Study on the Interaction between Lighting Level and Stimulus Spatial Location

**DOI:** 10.3389/fnhum.2015.00637

**Published:** 2015-11-24

**Authors:** Luis Carretié, Elisabeth Ruiz-Padial, María T. Mendoza

**Affiliations:** ^1^Facultad de Psicología, Universidad Autónoma de MadridMadrid, Spain; ^2^Departamento de Psicología, Universidad de JaénJaén, Spain

**Keywords:** event-related potentials (ERPs), visual stimuli, environmental light, photoreceptors, mesopic vision, central vision, peripheral vision

## Abstract

Due to heterogeneous photoreceptor distribution, spatial location of stimulation is crucial to study visual brain activity in different light environments. This unexplored issue was studied through occipital event-related potentials (ERPs) recorded from 40 participants in response to discrete visual stimuli presented at different locations and in two environmental light conditions, low mesopic (L, 0.03 lux) and high mesopic (H, 6.5 lux), characterized by a differential photoreceptor activity balance: rod > cone and rod < cone, respectively. Stimuli, which were exactly the same in L and H, consisted of squares presented at fixation, at the vertical periphery (above or below fixation) or at the horizontal periphery (left or right). Analyses showed that occipital ERPs presented important L vs. H differences in the 100 to 450 ms window, which were significantly modulated by spatial location of stimulation: differences were greater in response to peripheral stimuli than to stimuli presented at fixation. Moreover, in the former case, significance of L vs. H differences was even stronger in response to stimuli presented at the horizontal than at the vertical periphery. These low vs. high mesopic differences may be explained by photoreceptor activation and their retinal distribution, and confirm that ERPs discriminate between rod– and cone-originated visual processing.

## Introduction

The human –and other vertebrates– visual system counts with two types of retinal photoreceptors. Rods (which are ≈95% of photoreceptors (Jonas et al., 1992), are specialized in visual processing during darkness. Cones, the second type, are involved in daylight –or artificially equivalent– situations. Whereas rods and cones share common pathways to convey their signals toward the brain (Masland, 2001; Wässle, 2004), both types of photoreceptors diverge in the way they process our environment, and transmit differential information, due to their molecular bases and their retinal distribution (Curcio et al., 1990; Kawamura and Tachibanaki, 2008). Rods are distributed at extrafoveal retina, and cones are present throughout the whole retina, but their density is particularly high at the fovea and decreases with eccentricity. Thus, rod:cone anatomical ratio ranges from 1:1 at 0.4 mm eccentricity from central fovea to 30:1, approximately, at 10 mm eccentricity (Curcio et al., 1990). Interestingly, rod:cone anatomical ratio is smaller along the retinal meridian (the horizontal axis), than at the vertical axis (Curcio et al., 1990).

Vision may be classified as a function of rod and cone involvement. Thus, scotopic vision is characterized by an exclusive involvement of rods, and photopic vision by the exclusive involvement of cones. Between scotopic and photopic vision, a wide intermediate stage –mesopic vision–, especially interesting to study photoreceptor balance, combines rod and cone activity (Narisada and Schreuder, 2004; Stockman and Sharpe, 2006; Zele and Cao, 2014). Mesopic vision approximately ranges from starlight to twilight (Stockman and Sharpe, 2006), and is present in many indoor environments –including typical laboratories in which brain responses to visual stimuli are explored–. From the low threshold of mesopic vision (that shared with scotopic) to the high threshold (shared with photopic vision), there is a gradual change in the rod/cone functional bias from 100%/0% contribution to visual processing to 0%/100% (Schreuder, 2008).

Due to the differential spatial distribution of photoreceptors described above, brain activity in rod-biased and cone-biased light environments should heterogeneously differ as a function of the spatial location of the discrete visual stimuli being processed. Event-related potentials (ERPs), especially those recorded at occipital areas, have been reported to be sensitive to both the spatial location of the stimulation and the environmental light level. In the former case, several visual components of the ERPs, such as C1, P1, N1, P2, and N2, have shown a retinotopic pattern, changing their amplitude and even their polarity as a function of the spatial location of the discrete stimulus evoking it (e.g., Clark et al., 1994; Eimer, 1996; Di Russo et al., 2005). In the latter case, ERPs have manifested sensitivity to environmental light both in the frequency domain (Wooten, 1972) and in the amplitude domain (Münch et al., 2014). Indeed, several visual ERP components, such as P1 and P2 (among other components until ≈400 ms latency), have been suggested to show differential sensitivity to rod and cone activity (Cohn and Hurley, 1985; Rudvin and Valberg, 2006; Parisi et al., 2010; but the direction of these differences is controversial: e.g., P1 is reported as more sensitive to rod activity in the first study and to cone activity in the other two, see also divergent results on P2).

However, how the interaction between environmental light and spatial location of the stimulation modulates these amplitude, frequency and/or polarity effects has not been explored yet to the best of our knowledge. The scope of this study was to explore the effect of this interaction on brain –occipital– activity as measured through ERPs. To that aim, visual stimuli were presented at fixation (i.e., foveally projected) and out of fixation (i.e., peripherally projected) in low mesopic (≈0.03 lux) and high mesopic vision (≈6.5 lux), so whereas both rods and cones were active, their balance varied between both conditions. Our hypothesis was that, if ERPs are sensitive to rod– vs. cone-originated visual responses, low vs. high mesopic ERP differences should not be homogeneous, but they should vary as a function of the spatial location of the stimulus to be processed. Time, frequency and amplitude parameters –shown to be sensitive to environmental light and to spatial location, as indicated– were analyzed.

## Materials and Methods

### Participants

This study had been approved by the Research Ethics Committee of the Universidad de Jaén. Forty-two individuals, who provided their written informed consent, participated in this experiment, although data from only 40 of them could eventually be analyzed, as explained later (28 women, age range of 17–31 years, mean = 19.35, *SD* = 3.23). All participants were students of Psychology at the Universidad de Jaén and took part in the experiment voluntarily after providing informed consent. They reported normal or corrected-to-normal visual acuity.

### Stimuli and Procedure

**FIGURE 1:**
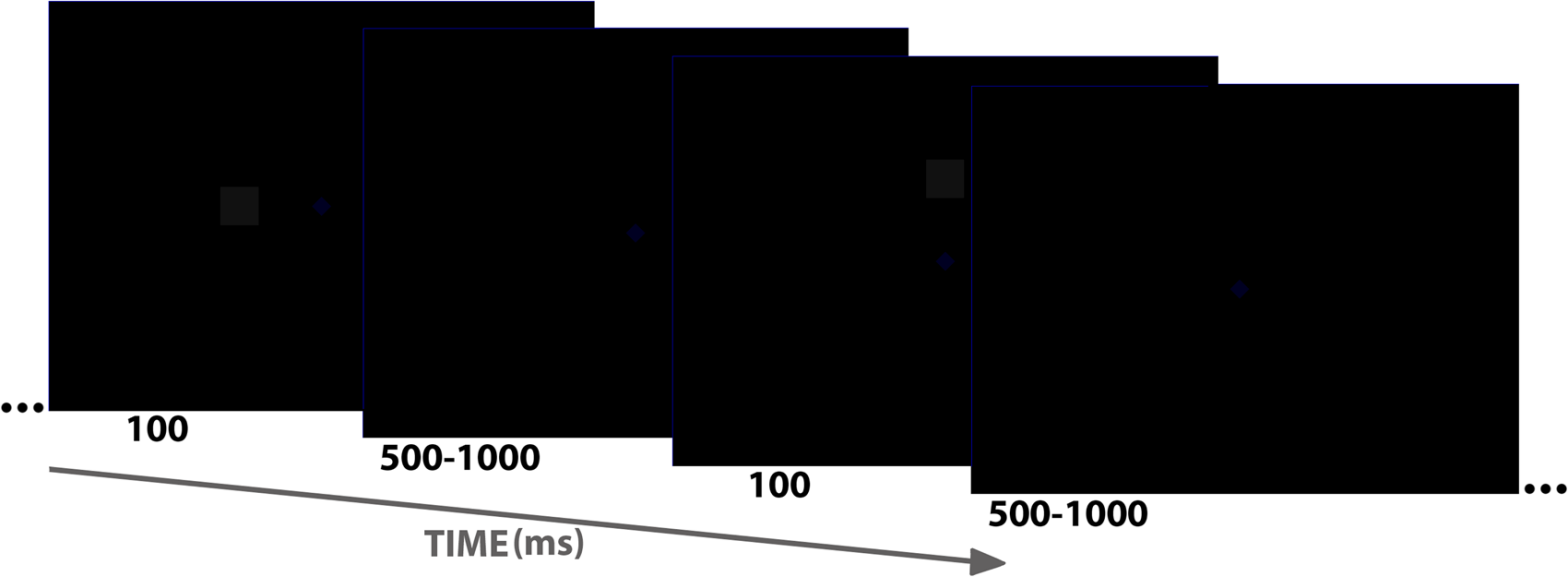
**Schematic representation of the stimulus sequence showing duration of stimuli and inter-trial interval as well as two of the five stimuli used (< or left to fixation and ∧ or above fixation).** Please note that both blue diamond (fixation) and gray square are very dark.

Participants were placed in an electrically shielded, sound-attenuated room, and stimuli were presented on a CRT screen (16 inches, 85 Hz). Their face distance from the screen was 60 cm. As shown in **Figure 1**, stimuli consisted of a black background (0, 0, 0 in the RGB scale, ranging from 0 to 256 in red, green and blue, respectively; 256, 256, 256 means absolute white), and two non-black small elements: a dark blue fixation diamond (0, 0, 34), and a dark gray square (17, 17, 17). Subjects were simply instructed to maintain the gaze toward the central fixation diamond (1.05° × 1.05°), which never disappeared, and to avoid blinking as much as possible. The square (2.2° side) was presented periodically (inter-trial interval –ITI– was aleatory ranging from 500 to 1000 ms; average ITI was 750 ms) for 100 ms at one of these locations: center (fixation or +), above fixation (∧), below (∨), left (<), and right (>). Visual angle with respect to fixation was the same in all cases: 4.77° from the center of the square to the center of the fixation diamond. Fifty trials for each of the five square locations were presented, yielding a total of 250 trials. The order of trials/locations was aleatory.

The Laboratory of Psychophysiology at the Universidad de Jaén, in which the experiment was run, has a double door access and no windows, so it allows for complete darkness. Recording sessions were all performed during day-light time. In the darker environment block (low mesopic, “L”), all lights in the laboratory were turned off with the exception of the screen with the task, so illuminance was ≈0.03 lux (as measured by an *Iso-Tech ILM 1337 light meter* placed in subject’ eyes and facing the light sensor toward the CRT screen while presenting a stimulus). In the lighter environment block (high mesopic, “H”), the adjustable light in the laboratory was set so illuminance measured in subjects’ eyes was ≈6.5 lux (measured in the same conditions). The same 250-trial run explained above was presented twice to subjects, one in the L block and the other in the H block (yielding 10 conditions: L+, L∧, L∨, L<, L>, H+, H∧, H∨, H<, H>). H and L environments were counterbalanced: 20 out of the 40 participants began with H, and the rest with L. Before each block, participants were asked to wait a 10 min adaptation period to light conditions prior to the beginning of the experimental run.

### Recording and Pre-processing

Electroencephalographic (EEG) activity was recorded using *BrainVision system* (Brain Products, Munich, Germany) with an electrode cap (*ElectroCap International*) with tin electrodes. Twenty-eight electrodes were placed on the scalp following a homogeneous distribution, but only occipital leads (placed at O1, Oz, and O2) were relevant for this study. All scalp electrodes were referenced to the nosetip. Electrooculographic (EOG) data were recorded supra- and infraorbitally (vertical EOG) as well as from the left vs. right orbital rim (horizontal EOG). An online analog bandpass filter of 0.3–40 Hz was applied. Recordings were continuously digitized at a sampling rate of 500 Hz. The continuous recording was divided into 600 ms epochs for each trial, beginning 100 ms before stimulus onset.

Ocular artifact removal was carried out in two steps. First, visual inspection of trials was carried out to eliminate any trial in which significant (>100 μV) ocular movements were detected (i.e., in which gaze moved from the fixation point). Second, blink-related interferences were removed through an independent component analysis (ICA)-based strategy (see a description of this procedure and its advantages over traditional regression/covariance methods in Jung et al., 2000), as provided in the *BrainVision Analyzer* software (Brain Products, Munich, Germany). This artifact rejection procedure led to the average admission of 48.75 trials per each of the 10 conditions (minimum = 43; *SD* = 0.14). One out of non-analyzed participants presented non-solvable anomalies in the recordings of one or more EEG critical leads (those at the occipital scalp), and the second was aleatorily discarded to reach a complete H/L counterbalance and to ensure the same male/female proportion (6/14) in both groups (i.e., H first and L first).

### Data Analysis

All analyses focused on occipital leads (O1, Oz, and O2), since, as explained in the Introduction, the occipital scalp region was our scope. A multiple analytical approach was designed in order to characterize differences among conditions in those parameters shown to be sensitive to environmental light and/or spatial location of the stimulation (see Introduction): amplitude differences as a function of time (ANOVAs on amplitudes at different time windows), time-series correlations (which further characterize amplitude and polarity differences), and frequency differences (ANOVAs on spectral densities). The Greenhouse–Geisser (GG) epsilon correction was applied to adjust degrees of freedom where necessary. Effect sizes were computed using the partial eta-square ($\eta_{p}^{2}$) method. *Post hoc* comparisons to determine the significance of pairwise contrasts were performed using the Bonferroni correction procedure.

These analyses followed an interval approach rather than a specific component approach for two reasons. On one hand, as explained in the Introduction, differential effects of photoreceptor activity are not circumscribed to specific components but have been reported in a wide range of latencies. On the other hand, as will be described later, ERP polarity inversion and phase variation as a function of experimental manipulations were systematic in this study, so labeling components as “Px” or “Nx” would become a spurious task.

#### Amplitude Analyses

Prior to statistical contrasts on amplitudes, L minus H subtractions were carried out across individual ERPs (participants × electrodes × stimulus spatial locations) in order to neutralize the marked main effect of spatial location of the stimuli on visual ERPs (e.g., Clark et al., 1994; this main effect was out of our scopes) and to emphasize the environmental light effect at each stimulus location. Windows of interest (WOIs) in these L minus H differences were established based on visual inspection of grand averages. Average (or area) amplitudes were quantified in each WOI, and their sensitivity to the spatial location of stimuli was tested through two repeated-measure ANOVAs: the ‘horizontal ANOVA’ measured the effect of the Horizontal Dimension (three levels: +, <, >), and the ‘vertical ANOVA’ tested the effect of the Vertical Dimension (three levels: +, ∧, ∨). Occipital Location (three levels: O1, Oz, O2) was introduced as the second factor in both ANOVAs. Vertical and Horizontal spatial dimensions were segregated in analyses since, as graphically shown later, WOIs differed between them.

#### Time-series Correlations

Event-related potentials were submitted to simple correlation analyses between H and L responses in order to further explore amplitude and polarity differences, as explained. These correlation analyses were carried out subject by subject, introducing H and L time series from 100 to 500 ms for each of the five stimulus locations (+, ∧, ∨, <, >) and in each of the three occipital recordings (O1, Oz, and O2). Also in this case, correlation coefficients were submitted to a Vertical (+, ∧, ∨) and to a Horizontal (+, <, >) ANOVA, Occipital Location (O1, Oz, O2) and Environmental Light (L, H) being also introduced as factors in both cases.

#### Frequency Analyses

Prior to frequency analyses, signals were submitted to a bandpass filter between 3 and 35 Hz since our interest was focused on the alpha rhythm, reported to be strongly associated with visual perception (Ergenoglu et al., 2004; Hanslmayr et al., 2007; Romei et al., 2008; van Dijk et al., 2008; Busch et al., 2009), neighbor rhythms –theta and beta– being also analyzed. Then, the power spectral density was calculated with a Hamming 400 ms window (100–500 ms) for each participant and occipital electrode in response to each stimulus location and light condition. These spectral analyses were performed, separately, for the theta band (4–8 Hz), alpha band (8–12 Hz) and beta band (12–30 Hz). Next, peak (maximal) spectral densities within each band were detected for each subject, condition and occipital electrode. Finally, these peak densities were submitted to two repeated measures ANOVAs for each frequency band (theta, alpha, and beta): as in previous analyses, the first ANOVA explored the Horizontal Dimension (+, <, >), and the second one explored the Vertical dimension (+, ∧, ∨). The other two factors introduced in both ANOVAs were Occipital Location (O1, Oz, O2) and Environmental Light (L, H).

## Results

**FIGURE 2:**
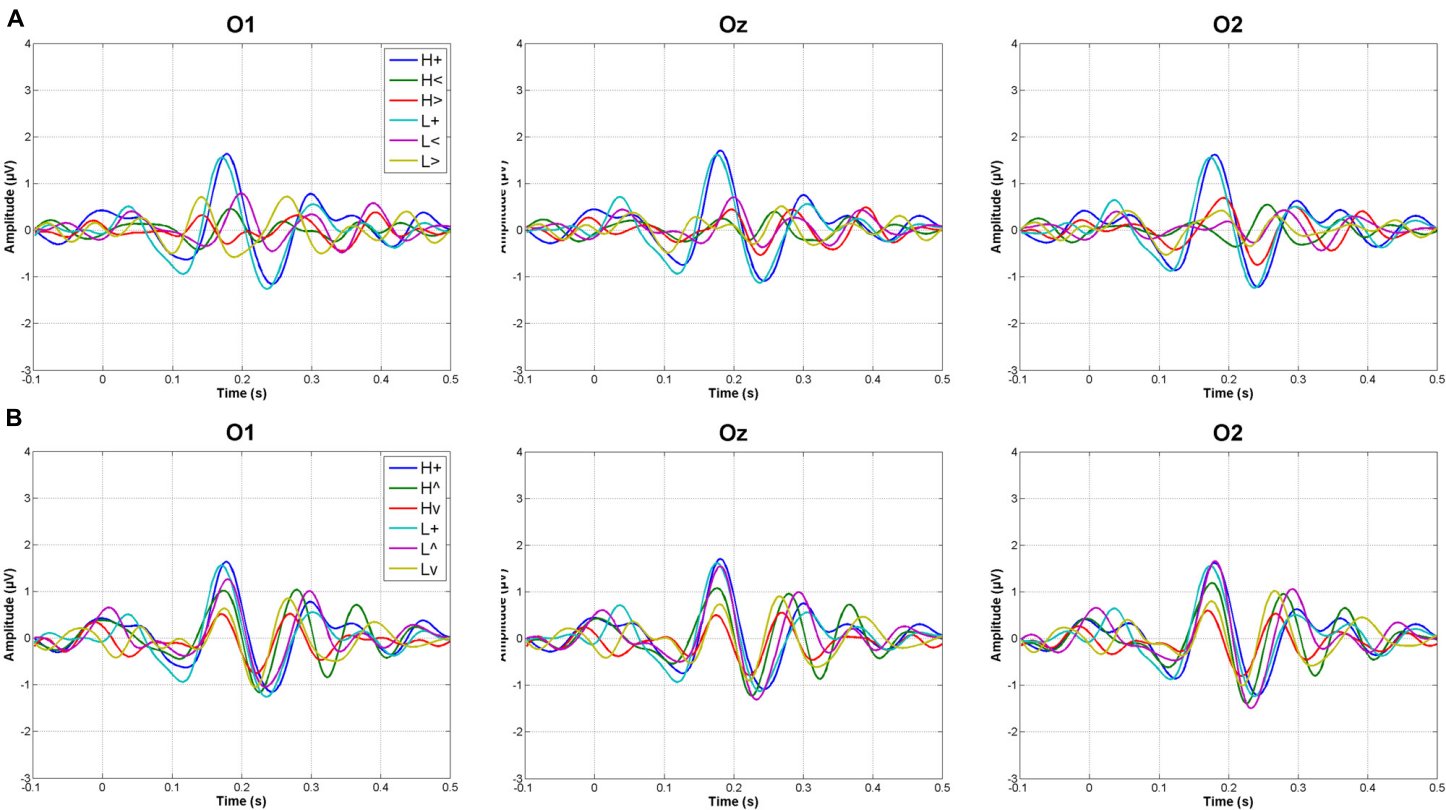
**Grand averages at occipital electrodes in response to vertically **(A)** and horizontally **(B)** distributed stimuli (L, low mesopic; H, high mesopic; +, stimuli presented at fixation; <, stimuli presented left; >, stimuli presented right; ∧, stimuli presented above fixation; ∨, stimuli presented below)**.

**Figure 2** shows grand averages of original recordings after subtracting the baseline (prestimulus) activity from each ERP. These grand averages correspond to occipital areas, which where those on which the study focused.

### Amplitude Analyses

**FIGURE 3:**
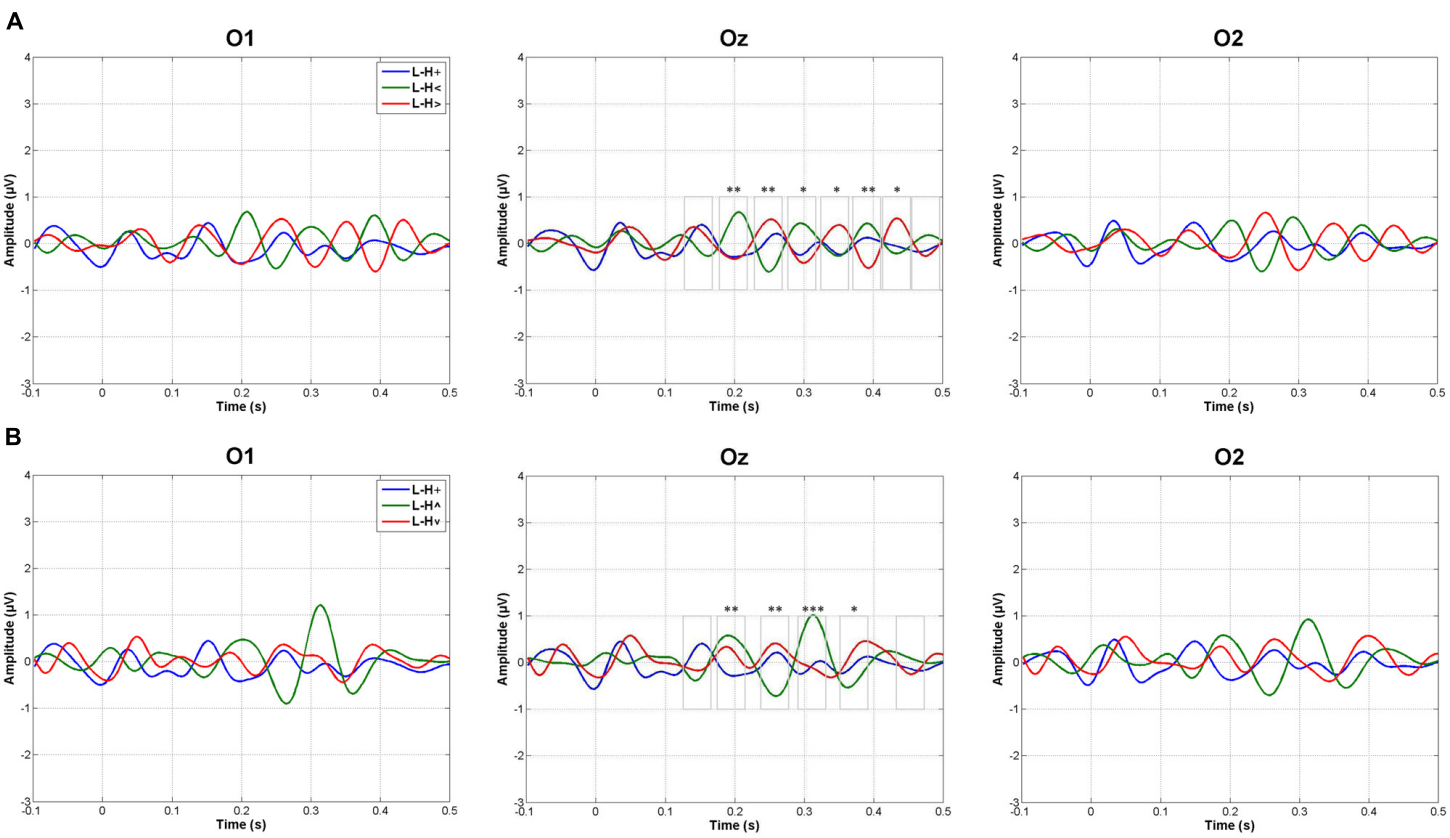
**Amplitude analyses.** Grand averages of low minus high mesopic (L–H) subtractions in response to **(A)** central and horizontal peripheral stimuli, and **(B)** in response to central and vertical peripheral stimuli (+, stimuli presented at fixation; <, stimuli presented left; >, stimuli presented right; ∧, stimuli presented above fixation; ∨, stimuli presented below). Windows of interest on which ANOVAs were carried out are also represented in Oz; one asterisk means a trend in ANOVAs (*p* < 0.1), two asterisks mean *p* < 0.05 significance, and three mean *p* < 0.005 significance: see **Table 1** and the main text.

**Figure 3** shows the grand averages of L minus H subtractions. As indicated in the Data Analysis section, WOIs for subsequent amplitude quantification were defined for those ERP intervals in which L minus H differences showed maximal values in grand averages. This criterion yielded eight WOIs in the case of Horizontal grand averages and six WOIs in the case of Vertical grand averages, as illustrated in **Figure 3** and numerically specified in **Table 1.** Both Vertical and Horizontal ANOVAs on these L minus H differences yielded significant differences as a function of spatial location at several WOIs between 140 and 450 ms approximately (**Table 1**; as may be appreciated, a trend –*p* < 0.1– was often found in the rest of WOIs). In general, greater L minus H amplitude differences were produced in response to peripheral stimuli than in response to stimuli presented at fixation (**Table 1**; **Figure 3**). Importantly, the pattern observed in the Horizontal dimension contrasts was symmetrical: no O1 vs. O2 differences were observed in any WOI.

**Table 1:** Statistical parameters associated to each window of interest (WOI) in Horizontal and Vertical ANOVAs on L minus H amplitudes (+, stimuli presented at fixation; <, stimuli presented left; >, stimuli presented right; ∧, stimuli presented above fixation; ∨, stimuli presented below; GG, Greenhouse–Geisser epsilon correction).

	WOI (ms)	*F*(2,78)	P (GG corrected)	$\eta_{p}^{2}$	*Post hoc* pairwise contrasts
Horizontal (<, +, >)	130–170	1.089	0.342	0.027	–
	180–220	4.714	0.012	0.108	< ≠ >, < ≠ +
	230–270	3.463	0.041	0.082	< ≠ >
	276–316	2.629	0.083	0.063	< ≠ >
	324–364	2.852	0.065	0.068	–
	370–410	3.601	0.034	0.085	< ≠ >
	416–456	2.616	0.083	0.063	–
	460–500	1.720	0.187	0.042	

Vertical (∧, +, v)	124–164	2.076	0.132	0.051	–
	174–214	3.459	0.038	0.081	–
	234–274	4.791	0.019	0.109	∧ ≠ ∨
	290–330	7.381	0.001	0.159	∧≠ ∨, ∧≠ +
	350–390	2.922	0.062	0.070	–
	430–470	1.343	0.267	0.036	–


### Correlations

**FIGURE 4:**
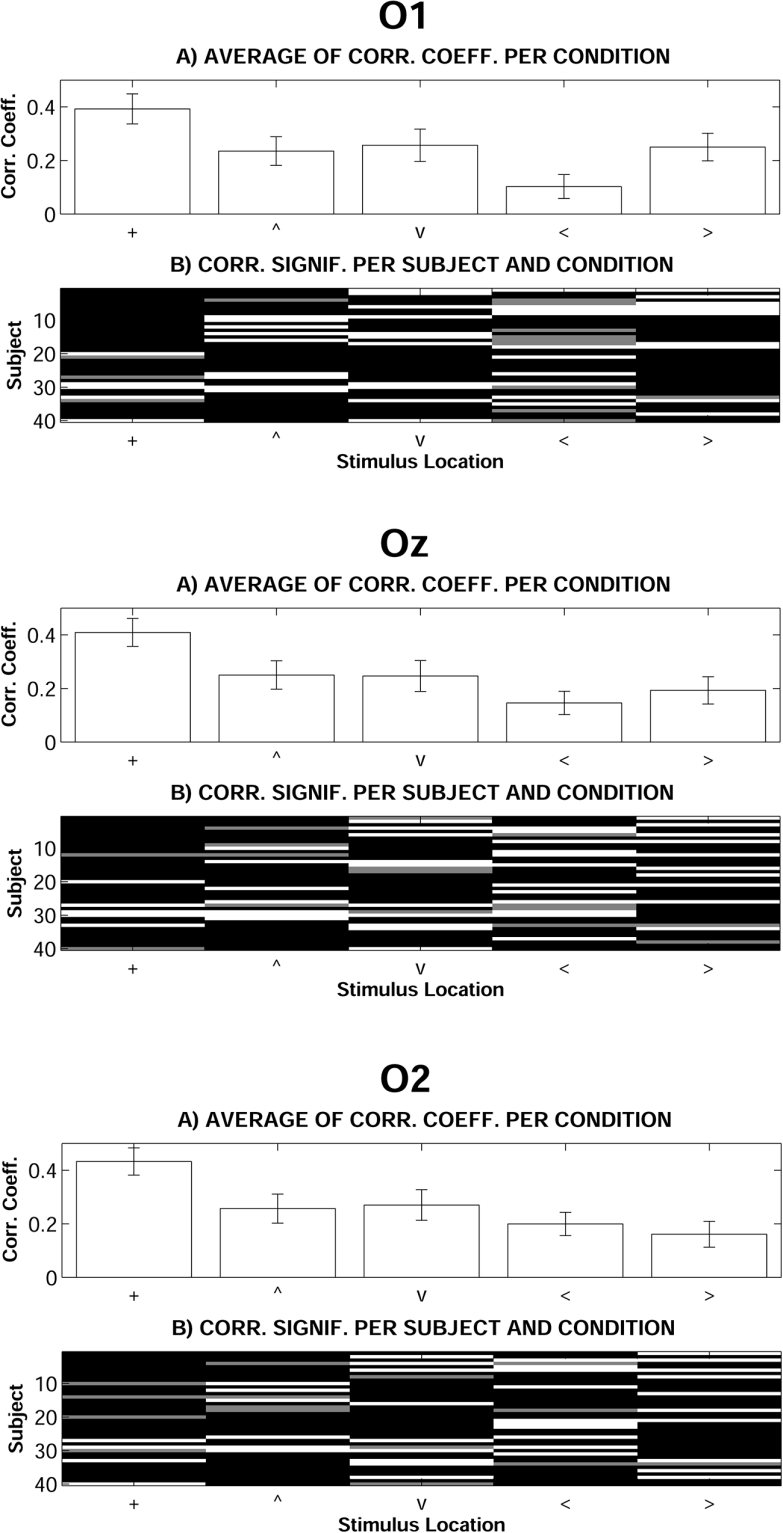
**Correlation analyses between Low and High mesopic conditions.** **(A)** Average of individual correlation coefficients (error bars indicate standard error of means). **(B)** Individual correlation significance: black means *p* < 0.01, gray means *p* < 0.05, white means non-significant (+, stimuli presented at fixation; <, stimuli presented left; >, stimuli presented right; ∧, stimuli presented above fixation; ∨, stimuli presented below).

Complementarily to previous analyses, and in order to test the extent to which H and L recordings were similar along the 100–500 time window, correlations were computed for each individual, occipital electrode and stimulus spatial location (see Data Analysis for details). **Figure 4** shows average correlation coefficients and case by case significances. Average correlation across participants between H and L recordings was significant (*p* < 0.05 in O1, Oz, and O2) only in response to stimuli presented at fixation. As also described previously, ANOVAs on L–H correlation coefficients for all stimulus spatial locations were computed. These analyses showed significant differences in the Vertical dimension [*F*(2,78) = 4.268, *p* < 0.05, $\eta_{p}^{2}$ = 0.099] and, more intensely, in the Horizontal dimension [*F*(2,78) = 13.980, *p* < 0.001, $\eta_{p}^{2}$ = 0.264]. In both cases, and according to *post hoc* contrasts, L–H correlation coefficients were significantly higher in response to stimuli presented at fixation than in response to <, >, and ∨ stimuli.

### Frequency Analyses

**FIGURE 5:**
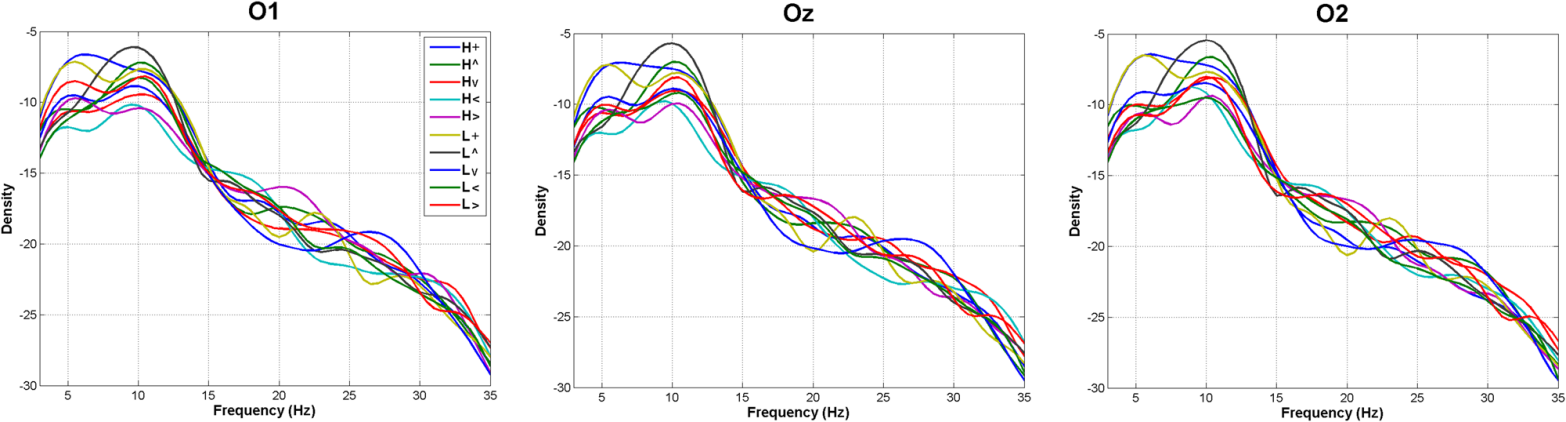
**Frequency analyses.** Average spectral densities in the Theta (4–8 Hz), Alpha (8–12 Hz), and Beta (12–30 Hz) bands (L, low mesopic; H, high mesopic; +, stimuli presented at fixation; <, stimuli presented left; >, stimuli presented right; ∧, stimuli presented above fixation; ∨, stimuli presented below).

**Figure 5** shows the spectrograms obtained for each of the 10 experimental conditions (environmental light × stimulus position). ANOVAs on peak spectral densities (see Materials and Methods) in the Theta, Alpha, and Beta bands were far from significance in any contrast involving main effects of Environmental Light, the interaction Environmental Light × Vertical dimension, or Environmental Light × Horizontal dimension (*p* > 0.4 in all cases), suggesting a similar H and L general spectral pattern whatever the spatial location of the stimulus was.

## Discussion

Brain occipital responses to the same stimuli in low mesopic (0.03 lux) and high mesopic (6.5 lux) environments presented important differences, and these differences were significantly modulated by the stimulus location. The experimental effects were not circumscribed to specific ERP components, but rather to a wide interval involving the majority of them, in line with findings showing multi-component –from P1 to P4–differential effects of photoreceptor activity (Cohn and Hurley, 1985; Rudvin and Valberg, 2006). The start of the interval was approximately 100 ms after the stimulus onset, which is in line with previous data –not exploring spatial location– reporting differential activity of occipital activity in response to visual stimuli presented during different environmental light conditions (Kojima and Zrenner, 1977; Münch et al., 2014). This latency seems therefore the beginning of the critical temporal window, which, in the present study, lasted up to ≈450 ms. Three results occurring within this temporal window may be underlined and may provide clues on the underlying mechanisms explaining the observed effects.

First, L (low mesopic, rod > cone activity balance) minus H (high mesopic, rod < cone balance) amplitude differences were much reduced in response to stimuli presented at fixation (foveally), while they were prominent in response to peripheral stimuli. This pattern was observed in several, periodic (∼10 Hz), temporal windows between 140 and 450 ms. This finding is reinforced by the fact that correlations between L and H responses showed that amplitude fluctuations along time (along the 100–500 ms window) were similar in H and L environments in response to foveally projected stimuli (correlations being significant in this case) but dissimilar in response to peripherally projected stimuli (correlations being non-significant). Second, L vs. H differences in response to peripheral stimuli were produced in both directions: alternatively, windows showed positive and negative L minus H differences (i.e., they alternatively showed L > H and L < H amplitudes). In other words, processes underlying the observed responses to peripheral stimuli should be complementarily active in both lighter and darker situations, and not only in one of them. And third, results suggest that differences may exist between the vertical and the horizontal spatial dimension as regards environmental light. Indeed, statistical contrasts showed stronger significance in L vs. H differences in response to stimuli presented at the horizontal periphery (left or right) than at the vertical periphery (up or down), although both were significant: **Figure 3.**

Distribution of photoreceptors in the retina fit well with these results and suggest that ERPs are indeed able to discriminate rod- from cone-originated visual responses. Rods are absent in the fovea, so stimuli presented at fixation mainly stimulate cones. As explained in the introduction, cones were involved in both L and H conditions. The involvement of a single type of photoreceptor in foveal processing could explain why H and L occipital responses to central stimuli correlated significantly. Whereas global cone signal would have been less intense in L than in H, sensory gain and visual adaptation mechanisms present throughout the visual pathway from the retina to the striate cortex (Kohn, 2007; Rieke and Rudd, 2009) would equalize L and H cone-originated signals at the visual cortex level (i.e., that recorded in ERPs). On the other hand, non-foveal retina presents both rod and cone photoreceptors (Curcio et al., 1990). As a consequence, the two types of photoreceptor intervened, but in opposite directions: non-foveal vision was more biased toward rod activity in L than in H, and more biased toward cone activity in H than in L. The fact that H vs. L amplitude differences were be larger in response to peripheral than to central stimuli, reinforces the idea that ERPs discriminate between cone- and rod-originated visual responses.

These ERPs differences between L and H lighting conditions were mainly characterized by a decoupled rhythm in response to peripheral stimuli: **Figure 3.** The decoupling began approximately at 140 ms (and remained up to 450 ms) and was especially regular (10 Hz) in response to the horizontal periphery (meridian), reflecting a clear counter-phasic occipital response pattern. Something similar, but to a lesser extent (counter-phase being not as regularly paced at a 10 Hz rhythm), occurred with respect to the vertical periphery. Peripheral decoupling was not due to frequency differences between light and dark situations: frequency analyses yielded no differences as a function of this factor. Providing an explanation to this phenomenon is difficult since no previous data exist, to the best of our knowledge, on a possible environmental light/darkness phase angles decoupling. However, it has been proposed that that the phase of ongoing brain oscillations, particularly in the alpha and theta frequency bands, modulate perception (Busch et al., 2009). Present results suggest that environmental light, and the type of photoreceptor involved, could affect the phase angles of EEG oscillations.

Interestingly, and as just mentioned, results show that H vs. L amplitude differences varied not only between fovea and periphery retinal projections, but also that certain divergence existed between vertical and horizontal periphery. The explanation could also lie in photoreceptor distribution. As indicated in the Introduction, rod:cone anatomical ratio is smaller along the retinal meridian (the horizontal axis), than at the vertical axis (Curcio et al., 1990). In other words, the meridian is the axis in which more balanced H/L photoreceptor activity should be produced. This could explain the more symmetrical –regularly counter-phasic, as explained– H vs. L pattern observed in response to stimuli presented in the horizontal periphery.

As a final remark, it is important to note that the heterogeneous photoreceptor distribution is also reflected in subsequent visual architecture. Thus, the visual route from retina to striate cortex consists of two parallel streams, the magnocellular and the parvocellular pathways. They originate from different retinal ganglion cells (Perry et al., 1984), which project to separate layers of the lateral geniculate nucleus (LGN) of the thalamus (Livingstone and Hubel, 1987). Critically, although rod and cone outputs join at the retinal ganglion cells level (Masland, 2001; Wässle, 2004), rod signals have been reported to be preferentially conveyed through the magnocellular pathway in humans and other primates (Purpura et al., 1988; Lee et al., 1997; Benedek et al., 2003). Therefore, high-order processes (i.e., originated at post-photoreceptor levels) involved in visual processing could also contribute to explain the observed effects. Interestingly, parvo vs. magnocellular action balance differentially affects visual ERP components such as N1 and P1 (e.g., Ellemberg et al., 2001; Hammarrenger et al., 2007).

In sum, this study demonstrates for the first time that ERPs are sensitive to the interaction between lighting condition and spatial location of visual stimulation. As indicated, the observed effects of this interaction were not circumscribed to specific ERP components, but rather to a wide interval (≈100–450 ms), involving the majority of them and including P1, N1, and P2, previously reported to be sensitive to photoreceptor activity and/or to the parvo vs. magnocellular balance, as indicated. Some suggestions can be advanced for future studies exploring this issue. First, including more extreme conditions (scotopic and/or photopic), along with mesopic vision, would serve to advance in the characterization of this interaction. Second, further methodological adjustments that may increase photoreceptor activity, such as increasing adaptation periods -10 min, in the present study- or manipulating the wave length of environmental light, would probably enhance the experimental effects observed here and provide additional relevant information. And third, whereas no frequency effects of environmental light were observed for alpha, beta, and theta bands, the analysis of gamma activity, often reported to be sensitive to diverse cognitive and affective processes (see reviews in Rieder et al., 2011 and Güntekin and Başar, 2014; respectively), would be of maximal interest. This would require and increased number of trials due to the low signal-to-noise ratio of gamma activity.

## Author Contributions

LC and ER-P have contributed equally to this study: experimental design, data analyses, and preparation of the manuscript (they are listed in alphabetical order). MM participated in experimental subjects recruiting and EEG data recollection.

## Conflict of Interest Statement

The authors declare that the research was conducted in the absence of any commercial or financial relationships that could be construed as a potential conflict of interest.
